# Editorial: Gene therapy for hearing loss: from mechanism to clinic, volume II

**DOI:** 10.3389/fnins.2024.1412981

**Published:** 2024-05-14

**Authors:** Shengyu Zou, Qingyin Zheng, Yu Sun, Xiaolong Fu, Wenjie Zhou, Zuhong He

**Affiliations:** ^1^Department of Otorhinolaryngology-Head and Neck Surgery, Zhongnan Hospital of Wuhan University, Wuhan, China; ^2^Department of Otolaryngology-Head and Neck Surgery, Case Western Reserve University, Cleveland, OH, United States; ^3^Department of Otorhinolaryngology, Union Hospital, Tongji Medical College, Huazhong University of Science and Technology, Wuhan, China; ^4^School of Life Sciences, Shandong Provincial ENT Hospital, Shandong University, Jinan, China; ^5^Songjiang Research Institute and Songjiang Hospital, Shanghai Jiao Tong University School of Medicine, Shanghai, China; ^6^State Key Laboratory of Digital Medical Engineering, Department of Otolaryngology Head and Neck Surgery, Zhongda Hospital, School of Life Sciences and Technology, Advanced Institute for Life and Health, Jiangsu Province High-Tech Key Laboratory for Bio-Medical Research, Southeast University, Nanjing, China

**Keywords:** hearing loss, hair cell, presbycusis, biomaterials, hearing regeneration, gene therapy

Hearing loss affects patients' quality of life and remains a major health problem worldwide due to the failure of damaged inner ear sensory cells to regenerate. This Research Topic presents advanced research progress in hearing. This research includes the application of advanced materials in the inner ear, a new method of hearing testing for congenital inner ear malformation in the clinic, research progress on inner hair cell regeneration and nursing care for deaf patients. This Research Topic introduces recent advances content in gene therapy for the inner ear disorder and describes cases of restored hearing through gene therapy. It also introduces some clinical issues including a new method of evaluating the structural disorder of inner ear through tympanogram, the activity changes of cerebral cortex area in sensorineural hearing loss and the treatment and nursing strategies of sudden sensorineural hearing loss. At the same time, this Research Topic also focuses on the impact of music on the psychological state of adolescents, and emphasizing the importance of fostering a beneficial music environment for adolescents. Meanwhile, this Research Topic introduces the latest research progress in materials science developing, including nanomaterials, magnetic responsive materials, and exosomes, and introduces their roles in the prevention and treatment of sensorineural hearing loss. This Research Topic also introduces some newly pathogenesis and intervention methods of sensorineural hearing loss. This Research Topic provides new ideas and methods for promoting the prevention and treatment of hearing loss.

Wang, Zheng et al. focused on recent advancements in gene therapy for cochlear hair cell (HC) regeneration, highlighting its potential to treat sensorineural hearing loss. These authors identified several transcription factors and downstream signaling pathways involved in HC development and regeneration. The abilities of several new gene editing therapies, CRISPR/Cas9 editing and viral vectors, to improve hearing recovery and HC regeneration are discussed. This review acknowledges the challenges in achieving effective HC regeneration and emphasizes the need for further research to optimize gene therapy approaches for clinical application.

Li A. et al. compared the difference in wideband acoustic immittance (WAI) between large vestibular aqueduct syndrome (LVAS) patients and the normal population. Compared with patients in the control group, patients in the LVAS group showed an increase in the overall absorbance of the tympanogram. Furthermore, the study compared the frequency-absorbance curves between LVAS patients and controls. The results revealed increased absorbance in the low- and medium-frequency ranges, suggesting that the maximum absorbance on the mean tympanogram could serve as a reliable indicator for evaluating inner ear structure disorders.

Liu et al. reviewed the progress in treating and nursing care for sensorineural hearing loss (SNHL) patients. Current clinical methods for treating SNHL include drug therapy, traditional Chinese medicine, hyperbaric oxygen therapy, and stem cell transplantation. The manuscript introduces some issues that needs paying attention in nursing care. It focuses on psychological care, dietary care, life care, medication care, and traditional Chinese medicine massage. Nurses could help patients establish a positive attitude to cooperate with treatment while also helping them develop healthy dietary plans and daily schedules. Nurses can also monitor potential side effects that patients may experience during treatment and promptly inform doctors to adjust treatment plans. As a characteristic medical treatment in China, they also introduced plans and nursing measures for patients with SNHL receiving traditional Chinese medicine treatment. This review stresses that ongoing research and patient-centered care are essential for advancing SNHL treatment and nursing practices.

Wang Y. et al. conducted a retrospective case–control study to investigate the impact of environmental noise exposure on the prognosis of sudden sensorineural hearing loss (SSNHL). The study compared patients with SSNHL exposed to environmental noise before onset (case group) to those with SSNHL without such exposure (control group). The study revealed that patients who were exposed to noise had a poorer prognosis, a longer duration of treatment, and a lower rate of vestibular dysfunction. The time to treatment and final pure-tone average (PTA) were significant prognostic factors, suggesting that early treatment and the severity of initial hearing loss are crucial in determining outcomes for SSNHL patients exposed to environmental noise.

Chen explored the influence of music on adolescent mental health, discussing both positive impacts, such as emotional expression and social bonding, and potential negative impacts, including the risk of hearing damage from excessive volume. First, they define music and introduce the classification and origin of music. Later, they discussed the benefits and drawbacks that music has to people. It suggests strategies for mitigating risks and enhancing benefits, emphasizing the importance of selecting appropriate music genres, creating a healthy music environment, advocating for positive music education, and encouraging active participation in music activities. This review aims to inform parents, educators, and community workers on fostering a beneficial music environment for adolescents.

Yang et al. provided a review of presbycusis, discussing its pathophysiology, genetic susceptibility, and impact of the environment on ARHL. Furthermore, they described the mechanisms that may induce ARHL, including ROS accumulation, inflammation and lateral wall fibrosis. Prevention strategies and current treatment options, including oral drugs and gene therapy targets, are also discussed. This paper emphasizes the need for further research to explore potential treatments such as antioxidants, anti-inflammatory agents and gene therapy.

Li J. et al. explored alterations in static and dynamic intrinsic brain activity in individuals with unilateral sudden sensorineural hearing loss (SSHL) using fractional amplitude of low-frequency fluctuation (fALFF) analysis. Significant differences in static fALFF patterns were detected between SSHL patients and healthy controls, revealing changes in brain functional areas and compensatory mechanisms in patients with SSHL. Dynamic fALFF analysis also revealed alterations, highlighting the importance of understanding neural changes in SSNHL patients for developing targeted interventions and rehabilitation strategies.

Fang et al. reviewed the use of advanced stem cells and nanomaterials for treating hearing loss, especially research advancements in combining nanomaterials with stem cells for hair cell regeneration. This manuscript describes the functions of various stem cells in hair cell regeneration. The role of exosomes, a newly discovered type of vesicle, in hearing has recently been explored. The author discussed the role of exosomes in hair cell regeneration and drug-induced hearing loss. The mechanism by which nanomaterials enhance the therapeutic effects of stem cells and the promising results of stem cell-derived exosomes in tissue repair are discussed. Despite technical and practical limitations, the findings are encouraging for future clinical applications, highlighting the need for continued research on stem cell therapy for hearing loss.

Wang, Deng et al. reviewed advancements in stem cell (SC) therapy for regenerative medicine. This review describes the classification and function of SCs and the regulatory mechanisms of SCs. In the field of SC therapy, researchers have focused on the gene regulatory mechanism of SCs and the therapeutic approach for SCs transplanted via vectors. The review covered various types of stem cells, including their characteristics, potential applications in cell and cell-free therapies, and technical routes of therapy. This review addresses current challenges in the field, such as safety issues and differentiation control, and highlights the significant therapeutic potential of stem cells in treating a wide range of diseases and traumatic conditions.

Feng et al. reviewed recent advances in the genetic etiology of non-syndromic deafness in children, focusing on the high incidence of genetic hearing loss. This study aimed to assist in personalized diagnosis and treatment by summarizing key findings in genetic research related to non-syndromic deafness. This paper discusses various genes implicated in hearing loss, the pathophysiology, and the molecular mechanisms underlying this condition. This finding emphasized the importance of genetic testing and screening in developing innovative treatment and management strategies for affected children.

Sun et al. explored the potential application value of pigment epithelium-derived factor (PEDF) in treating sensorineural hearing loss (SNHL). As a protein with cellular protection and antioxidative properties, PEDF has protective effects on inner hair cells and promotes cell differentiation. Subsequently, they analyzed the signaling pathway by which PEDF promotes antioxidant, anti-inflammatory and cell differentiation. This highlights the multifunctional role of PEDF, including its cellular protection and antiangiogenic properties, which might offer new treatment options for inner ear diseases. This review assessed the performance of PEDF in treating SNHL, its mechanisms, and therapeutic prospects, suggesting that further research and clinical trials are needed to establish its safety and effectiveness in treating inner ear disease.

Zhu et al. provided a comprehensive overview of the role of non-coding RNAs (ncRNAs), particularly microRNAs (miRNAs) and long non-coding RNAs (lncRNAs), in the pathogenesis of hearing loss. They described the role of miRNAs in various types of hearing loss, including age-related hearing loss, drug-induced hearing loss, noise-induced hearing loss, and the regulation of hair cell regeneration. These findings emphasize the significance of ncRNAs in regulating various physiological and pathological processes that impact hearing loss development and prognosis. This review discussed the potential of ncRNAs as therapeutic targets for precise treatment strategies, addressing the current challenges and future prospects in the study of ncRNAs related to hearing loss.

Lu et al. reviewed the current advances in biomaterials for inner ear cell regeneration, focusing on their application in constructing physiologically relevant 3D culture systems that mimic the stem cell microenvironment. The use of various biomaterials, including hydrogels, conductive materials, magnetoresponsive materials, and photomodulation materials, is highlighted because of their potential to support the regeneration and functional maturation of inner ear cells. This review emphasized the importance of selecting and combining biomaterials strategically based on their physicochemical properties to overcome challenges in inner ear cell regeneration research.

Liu and Xu discussed the role of macrophage-related immune responses in sensorineural hearing loss (SNHL), emphasizing their potential as therapeutic targets. This review described the origin and distribution of inner ear macrophages. Furthermore, they analyzed macrophage activation during acute and chronic cochlear injury. The dynamic changes in macrophages in various inner ear injuries were reviewed, and their potential role in mitigating damage was clarified. This review suggested that gene therapy targeting immune responses could be a promising direction for reconstructing hearing.

For this Research Topic, research on gene therapy in the fields of sensorineural hearing loss, inner ear stem cells, new biological materials, tissue engineering, and other new technologies and methods of development was performed. The content of this Research Topic includes recent research progress in the auditory system and the latest inner ear gene therapy methods and thus could provide a reference for further exploration of auditory disorders.

## Author contributions

SZ: Writing – original draft. QZ: Writing – review & editing. YS: Writing – review & editing. XF: Writing – review & editing. WZ: Writing – review & editing. ZH: Writing – original draft, Writing – review & editing.

